# The diagnostic value of contrast-enhanced ultrasonography in breast ductal abnormalities

**DOI:** 10.1186/s40644-023-00539-w

**Published:** 2023-03-10

**Authors:** Bo Wang, Di Yang, Xuan Zhang, XuanTong Gong, Tong Xu, Jie Han, YinPeng Ren, ShuangMei Zou, Lin Li, Yong Wang

**Affiliations:** 1 Department of Ultrasound, National Cancer Center, National Clinical Research Center for Cancer, Cancer Hospital, Chinese Academy of Medical Sciences and Peking Union Medical College, Beijing, 100021 China; 2 Department of Breast Surgery, National Cancer Center, National Clinical Research Center for Cancer, Cancer Hospital, Chinese Academy of Medical Sciences and Peking Union Medical College, Beijing, 100021 China; 3 Department of Pathology, National Cancer Center, National Clinical Research Center for Cancer, Cancer Hospital, Chinese Academy of Medical Sciences and Peking Union Medical College, Beijing, 100021 China; 4 Department of Radiology, National Cancer Center, National Clinical Research Center for Cancer, Cancer Hospital, Chinese Academy of Medical Sciences and Peking Union Medical College, Beijing, 100021 China

**Keywords:** Breast, Ultrasonography, Contrast-enhanced ultrasonography, Ductal abnormalities

## Abstract

**Background:**

Ductal lesions are an important, often overlooked, and poorly understood issue in breast imaging, which have a risk of underlying malignancy ranging from 5 to 23%. Ultrasonography (US), which has largely replaced galactography or ductography, has become an important imaging method to assess patients with ductal lesions. However, it is difficult to distinguish benign from malignant ductal abnormalities only by ultrasonography, most of which are recommended to be at least in subcategory 4A; these require biopsy according to the ACR BI-RADS®atlas 5th Edition-breast ultrasound. Contrast-enhanced ultrasound (CEUS) has been shown to be valuable for differentiating benign from malignant tumors, but its value is unclear in breast ductal lesions. Therefore, the purposes of this study were to explore the characteristics of malignant ductal abnormalities on US and CEUS imaging and the diagnostic value of CEUS in breast ductal abnormalities.

**Methods:**

Overall, 82 patients with 82 suspicious ductal lesions were recruited for this prospective study. They were divided into benign and malignant groups according to the pathological results. Morphologic features and quantitative parameters of US and CEUS were analyzed by comparison and multivariate logistic regression to determine the independent risk factors. The diagnostic performance was assessed by receiver operating characteristic (ROC) curve analysis.

**Results:**

Shape, margin, inner echo, size, microcalcification and blood flow classification on US, wash-in time, enhancement intensity, enhancement mode, enhancement scope, blood perfusion defects, peripheral high enhancement and boundary on CEUS were identified as features correlated with malignant ductal lesions. However, multivariate logistic regression showed that only microcalcification (OR = 8.96, *P* = *0.047*) and enhancement scope (enlarged, OR = 27.42, *P* = *0.018*) were independent risk factors for predicting malignant ductal lesions. The sensitivity, specificity, positive predictive value, negative predictive value, accuracy and area under the ROC curve of microcalcifications combined with an enlarged enhancement scope were 0.895, 0.886, 0.872, 0.907, 0.890, and 0.92, respectively.

**Conclusions:**

Microcalcification and enlarged enhancement scope are independent factors for predicting malignant ductal lesions. The combined diagnosis can greatly improve the diagnostic performance, indicating that CEUS can be useful in the differentiation of benign and malignant lesions to formulate more appropriate management for ductal lesions.

**Supplementary Information:**

The online version contains supplementary material available at 10.1186/s40644-023-00539-w.

## Background

Ductal lesions are an important, often overlooked, and poorly understood issue in breast imaging and commonly represent a variety of benign entities, including duct ectasia, fibrocystic changes, mastitis, fibroadenomas, intraductal debris, intraductal papilloma, and malignant invasive or in situ ductal carcinoma [1]. In patients with ductal lesions, it is of primary importance to exclude the presence of a malignant lesion that requires immediate excision [2]. For benign lesions, especially intraductal papilloma, many recent studies have shown that follow-up observation rather than surgical excision may be necessary [3–6]. Therefore, the management of benign and malignant lesions is completely different: surgical excision or imaging follow-up, which largely depends on the accurate diagnosis of benign and malignant lesions.

However, the first-line imaging technique of mammography often does not finely portray these lesions and has low sensitivity [7, 8]. Galactography was previously considered the diagnostic procedure of choice in patients with ductal lesions, but it is rarely performed due to its invasiveness and potentially related complications [9, 10]. MRI is a relatively good method to evaluate breast ductal lesions, but it is expensive and time-consuming [11].

Recent advances in ultrasound, especially scanning with a high-frequency transducer, have enabled the clear demonstration of duct systems, making it possible to obtain images of small intraductal lesions [12–14]. Ductal lesions usually manifest as ductal abnormalities on US imaging, which is one of the findings often encountered in US examination. In ACR BI-RADS ATLAS, “ductal abnormalities” are called duct changes included in “associated features” [15]. Few studies have investigated US findings of duct abnormalities, and no morphological criteria in the BI-RADS US lexicon are recommended to indicate malignant ductal lesions. Therefore, it remains difficult to describe and manage these lesions.

Recently, contrast-enhanced ultrasound (CEUS) was proven to be an effective way of differentiating benign from malignant breast tumors [16–18]. The shape, boundary and other findings of the abnormalities can be clearly shown by CEUS. Additionally, microcirculation perfusion inside the tumor presented by CEUS can provide more diagnostic information to conduct qualitative and quantitative analysis of the lesions [16]. Therefore, it can be reasonably assumed that different characteristics of enhancement reflect different microcirculation perfusion of the lesions, which can provide more details to distinguish malignant and benign ductal lesions. Importantly, no prior study has identified CEUS for its diagnostic value in breast ductal lesions.

In this study, we aimed to investigate the characteristics of US and CEUS in breast ductal lesions. Furthermore, we focused on the value of CEUS for differentiating benign from malignant ductal lesions to formulate a more appropriate management plan, decrease the cost of medical care and better manage ductal lesions.

## Patients and methods

### Patient selection

**Fig. 1:**
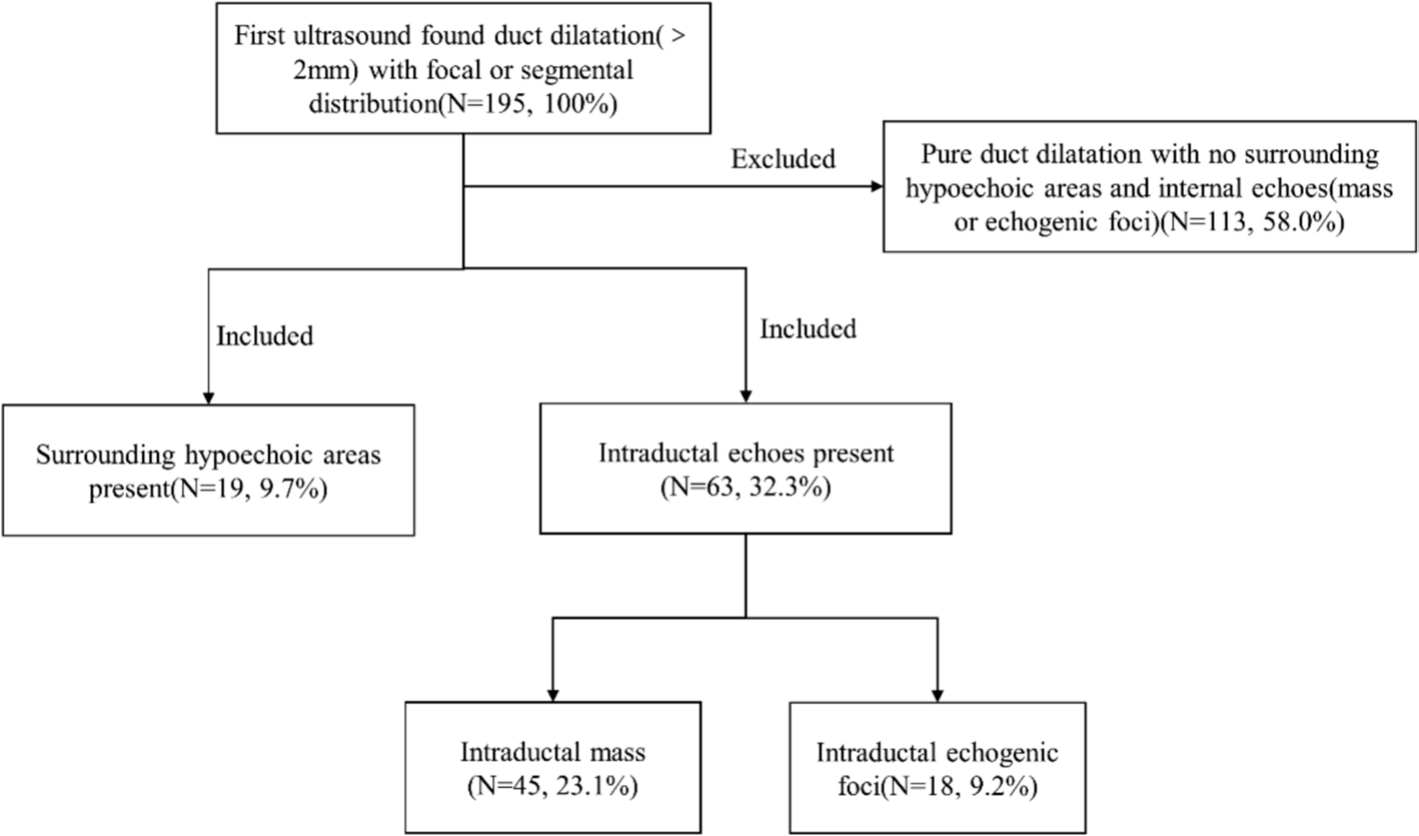
Flowchart of patients (included, excluded and the percentage)

Patient selection came from outpatient and breast ultrasound screening patients. Outpatient patients suspected of breast ductal lesions due to nipple discharge were examined by ultrasound. Breast ultrasound screening patients had ductal abnormalities on ultrasound with or without related symptoms. The ultrasound examination (before mammography) manifesting duct dilatation (> 2 mm) with focal or segmental distribution was defined as ductal abnormalities [15, 19]. Segmental distribution refers to the distribution consistent with the mammary duct system, and focal distribution indicates that it is restricted to a certain area. A total of 195 cases manifested duct dilatation (> 2 mm) with focal or segmental distribution on ultrasound examination; 113 cases were excluded because they showed pure duct dilatation with no surrounding hypoechoic areas and intraductal echoes. Nineteen cases showing duct dilatation with surrounding hypoechoic areas, 45 cases showing intraductal masses and 18 cases showing intraductal echogenic foci were included. Therefore, a total of 82 consecutive patients with 82 suspicious ductal lesions in this prospective study were recruited in our institution from January 2019 to December 2019. A flowchart of patients (included, excluded and the percentage) is shown in Fig. 1. This study was approved by the Ethics Committee of our institution, and informed oral and written consent was obtained from all patients.

### The inclusion criteria

**Fig. 2:**
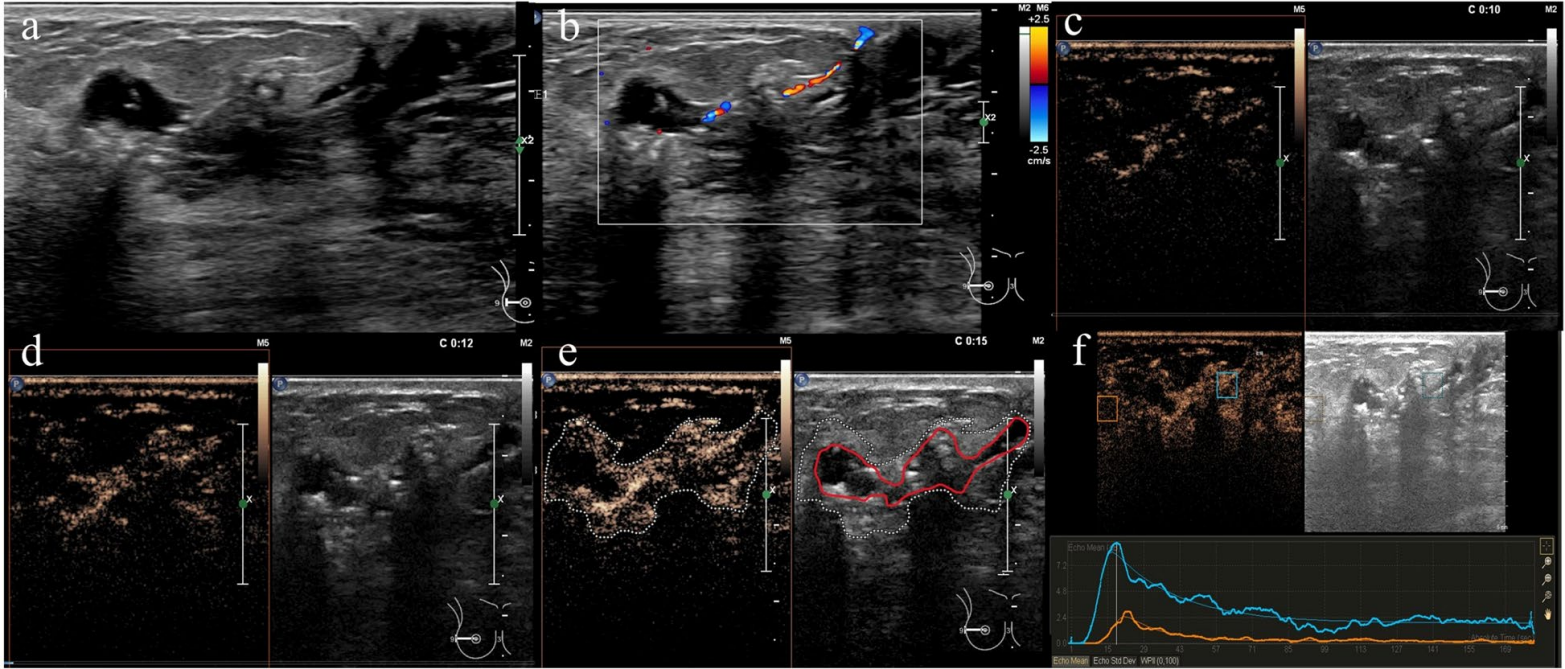
A 61-year-old woman with ductal carcinoma in situ with 20% invasion. Conventional US showing a segmental duct dilatation area with microcalcification (echogenic foci) in the retroareolar region (**a**). A stripe blood flow signal can be seen on CDFI (**b**). CEUS showing heterogeneity and hyperenhancement along the duct (**c**, **d**, **e**); its enhancement scope (e dotted line area) was significantly larger than that of conventional US (red line area). The time-intensity curve shows fast in and fast out compared with the surrounding normal breast tissues (**f**)

**Fig. 3:**
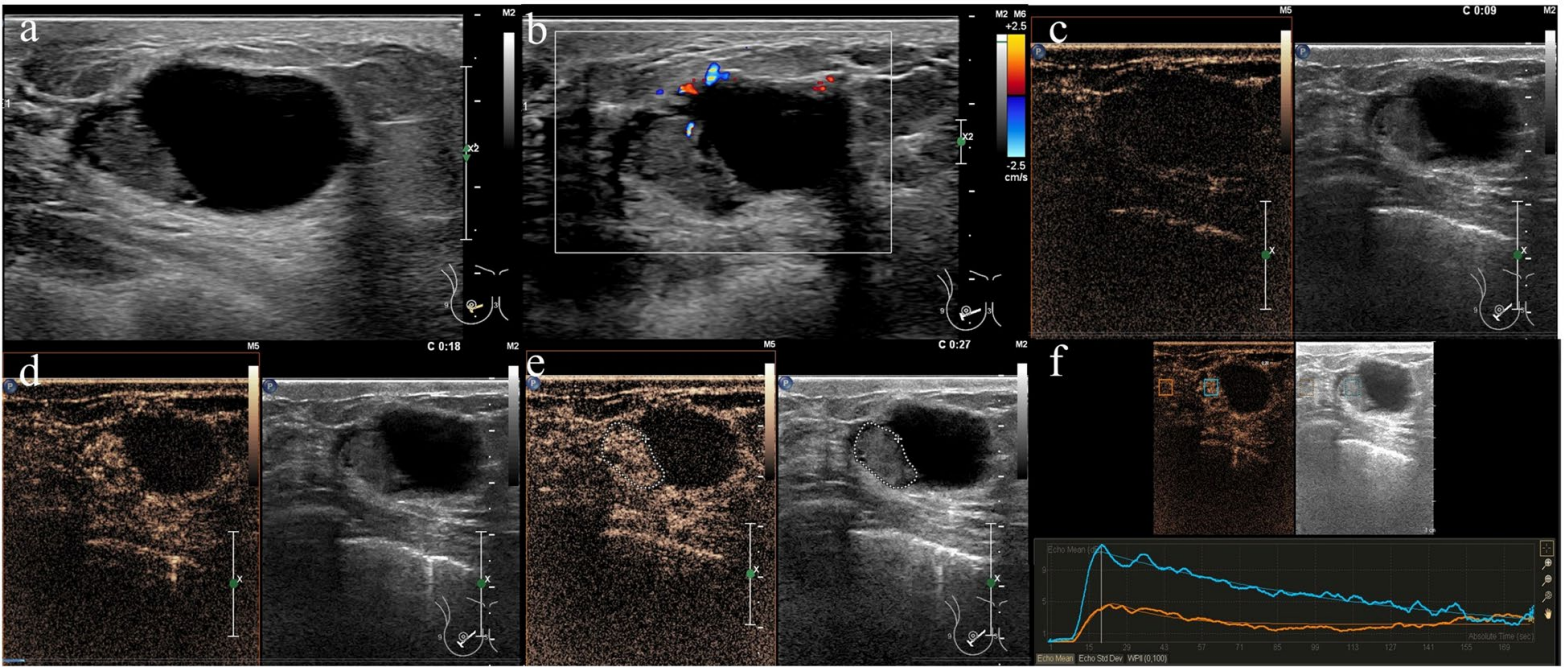
A 42-year-old woman with intraductal papilloma. Conventional US showing a well-circumscribed mass in the dilated duct (**a**). Dotted blood flow signal in the mass can be seen on CDFI (**b**). CEUS showing homogeneous and hyperenhancement (**c**, **d**, **e**); its enhancement scope was not enlarged compared with conventional US (E dotted line). The time-intensity curve shows that blood perfusion is fast in and fast out compared with the surrounding normal breast tissues (**f**)

1. Duct dilatation with surrounding hypoechoic areas (Fig. 2a); 2. Intraductal calcification (echogenic foci (Fig. 2a)); 3. Intraductal masses (Fig. 3a).

### The exclusion criteria

1. Pure duct dilatation with no surrounding hypoechoic areas and internal echoes;
2. Patients with mental abnormality, severe multisystem failure and those who failed to cooperate with the examination;
3. Pregnancy and any other contraindications to CEUS.

### US and CEUS examination

US and CEUS examinations were performed using a high-frequency (5–12 MHz) linear-array transducer (Philips EPIQ5). SonoVue (Bracco Suisse SA, Geneva, Switzerland) was selected as the contrast agent. Details of the US and CEUS examination processes are described in Supplementary materials 1 and 2.

### Imaging analysis

All of the examinations and imaging analyses were conducted by 2 radiologists with at least 5 years of experience in breast US and CEUS. A third radiologist with more than 10 years of experience intervened if a consensus could not be reached. All of them were blinded to the chart reviews of the patients.

Grayscale characteristics, including size, shape, margin, inner echo, posterior features, microcalcification, and blood flow classification, were recorded during US assessment. The microcalcification in this study was identified as echogenic foci located within a duct or surrounding hypoechoic areas, distributed in a branch or cluster pattern or restricted to a certain area. Mammography (MG) was used to confirm whether the echogenic foci seen on US were microcalcifications. Therefore, for mammographic (MG) images, we only focused on calcification in this study. According to the grades of Adler, blood flow classification of the Doppler ultrasound color flow imaging was divided into 4 categories (Supplementary material, 3).

Quantitative and qualitative characteristics of CEUS images or videos were evaluated. The qualitative characteristics of the lesions included: (a) wash-in/wash-out patterns; (b) enhancement intensity: hyperenhancement, isoenhancement or hypoenhancement; (c) texture of enhancement: homogeneous, heterogeneous; (d) boundary: clear or unclear; (e) enhancement scope: enlarged or not enlarged, which is compared with greyscale US. Enhancement scope is the area where the lesion is enhanced compared with the surrounding breast parenchyma (For example, Figs. 2e/ 3e/ 4e dotted line area); (f) perilesional enhancement: presence or absence; (g) perfusion defects: presence or absence; (h) radial-penetrating vessels defined as vessels from surrounding tissue toward the lesion: presence or absence.

**Fig. 4:**
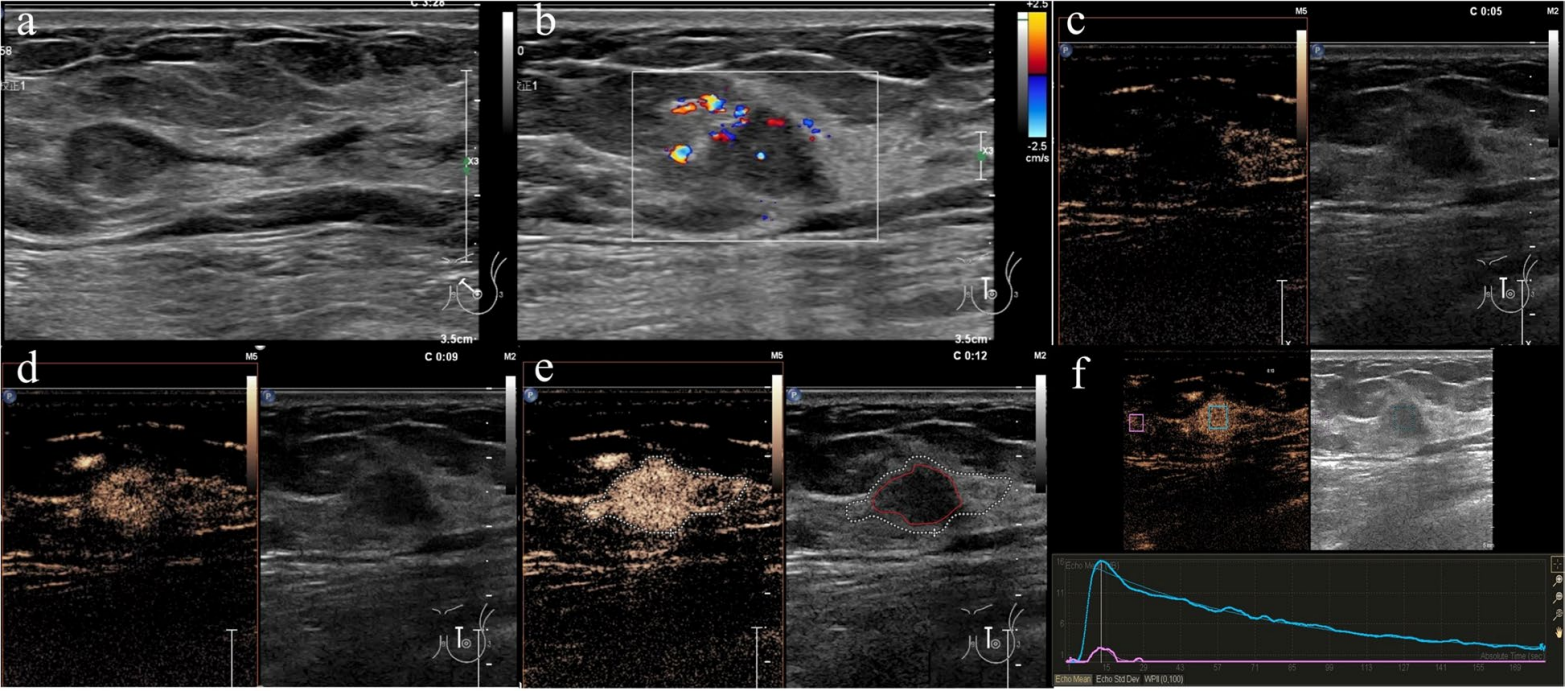
A 56-year-old woman with intraductal papillary carcinoma. Conventional US showing a well-circumscribed oval mass connected to the dilated duct (**a**). Strip and dotted blood flow signals can be seen on CDFI (**b**). CEUS showed heterogeneity and hyperenhancement in this mass (**c**, **d**, **e**), and its enhancement scope (E dotted line area) was significantly larger than that of conventional US (red line area). The time intensity curve shows fast in and fast out (**f**)

CEUS quantification software (QLAB; Philips, GeR) was used to obtain quantitative parameters. The CEUS video was analyzed through frame-by-frame playback. The most perfused region was selected as the region of interest (ROI) in each lesion. The time-intensity curve was subsequently obtained, including arrival time (AT), time to peak (TTP), peak intensity (PI), rising slope (k), area under the curve (AUC), and mean transit time (MTT). The definition of these parameters is shown in Supplementary materials 4.

BI-RADS classification of US was conducted according to ACR 2013 ultrasound BI-RADS (5th edition).

### Histopathology

The histopathology of the 82 breast ductal lesions was confirmed by US-guided core needle biopsy (*n* = 22) or surgical resection (*n* = 60). All lesions were divided into a benign group and a malignant group according to the pathological results of surgical resection or biopsy.

### Statistical analysis

For continuous data, the Kolmogorov–Smirnov test was used to check the normal distributions. Normally distributed data used the independent-sample t test, while the nonnormally distributed variables used the Mann–Whitney U test. Interobserver variability was analyzed by Kappa test. Pearson’s χ^2^ test or Fisher’s exact test, when appropriate, was used to compare categorical data. The independent risk factors for malignant ductal lesions were identified through binary logistic regression analysis. Subsequently, a receiver operating characteristic curve analysis was conducted to assess the diagnostic performance of each variable that was statistically significant in the regression analysis model. Statistical significance was considered as a two-tailed *P* value < *0.05*. All statistical analyses were performed using SPSS (Version 23.0; Armonk, New York).

## Results

### Clinical data

A total of 82 patients with 82 suspicious ductal lesions fulfilled the selection criteria. All patients were female. The average age of the malignant group was older than that of the benign group (*P* = *0.004*). The most common symptoms were breast pain/discomfort and a palpable breast mass; the second most frequent presentation was nipple discharge (Table 1). The pathological results showed that malignancy was present in 38 patients: 27 ductal carcinoma in situ (DCIS), 5 intraductal papillary carcinoma, 5 infiltrating ductal carcinoma, and 1 mucinous adenocarcinoma. Benign lesions were present in 44 patients: 22 intraductal papilloma, 6 fibroadenoma, 10 adenosis, 3 intraductal debris, 2 usual ductal epithelial hyperplasia, and 1 fat necrosis.

**Table 1:** Clinical characteristics of ductal lesions

Characteristics	All patients	Benign, n (%) (*n* = 44)	Malignant, n (%) (*n* = 38)	**χ** ^2^/t	*P*
Age(y)	48.7 ± 9.5	46.0 ± 7.8	51.9 ± 10.4	-2.944	0.004*
Symptoms					
Pain/discomfort and palpable	36	11	25	0.033	0.535
Nipple discharge	28	23	5		
None	18	10	8		

^*^Represents *P* < *0.05*

### US findings

The shape (*P* < *0.001*), margin (*P* < *0.001*), inner echo (*P* < *0.001*), size (*P* = *0.009*), microcalcification (*P* < *0.001*) and blood flow classification (*P* = *0.002*) were significantly different between the benign and malignant groups (Table 2). 2). The mean size of the malignant lesions was larger than that of benign lesions (*P* = *0.009*). An irregular shape, uncircumscribed margin, and heterogeneous inner echo were the most frequent findings in malignant lesions. Microcalcification was more frequently detected in malignant lesions than in benign lesions (16/38, 42.1% *vs.* 2/44, 4.5%, *P* < *0.001*).

**Table 2:** Comparative analysis of conventional US characteristics of benign and malignant ductal lesions

Characteristics	Benign, n (%) (*n* = 44)	Malignant, n (%) (*n* = 38)	**χ** ^2^/t	*P*
Shape			16.067	< 0.001^*^
Regular	26(59.1)	6(15.8)		
Irregular	18(40.9)	32(84.2)		
Margin			19.953	< 0.001^*^
Circumscribed	31(70.5)	8(21.1)		
Not circumscribed	13(29.5)	30(78.9)		
Inner echo			14.024	< 0.001^*^
Homogeneous	26(59.1)	7(18.4)		
Heterogeneous	18(40.9)	31(81.6)		
Size(cm)	1.43 ± 0.84	1.98 ± 0.99	-2.684	0.009^*^
Posterior feature			1.076	0.580
None	42(95.5)	34(89.5)		
Enhancement	1(2.3)	2(5.3)		
Shadowing	1(2.3)	2(5.3)		
Blood flow classification			12.775	0.002^*^
0	15(34.1)	5(13.2)		
1	24(54.5)	16(42.1)		
2&3	5(11.4)	17(44.7)		
Microcalcifications^a^			16.791	< 0.001^*^
Presence	2(4.5)	16(42.1)		
Absence	42(95.5)	22(57.9)		
MG				
Calcifications	15(34.1)	23(60.5)		
No calcification	29(65.9)	15(39.5)		

^a^The microcalcifications seen on US were confirmed by mammography (MG). * *P* < *0.05*

### CEUS findings

CEUS images indicated that 4 lesions (3 intraductal debris and 1 mucinous adenocarcinoma) showed no blood perfusion. CEUS images indicated blood perfusion in the remaining 78 lesions. Wash-in time (*P* < *0.001*), enhancement intensity (*P* < *0.001*), enhancement mode (*P* = *0.017*), enhancement scope (*P* < *0.001*), blood perfusion defects (*P* < *0.001*), peripheral high enhancement (*P* < *0.001*) and boundary (*P* < *0.001*) were significantly different between the benign and malignant groups (Table 3). The most frequent CEUS characteristics of malignant ductal lesions were fast (wash-in earlier and with a shorter time to peak compared with surrounding breast parenchyma) enhancement (89.2%), heterogeneous enhancement (73%), high (higher intensity of enhancement compared with surrounding breast parenchyma) enhancement (86.5%), unclear boundary (75.7%) and enlarged enhancement scope (78.4%). The enlarged enhancement scope was more common in malignant lesions than benign lesions (29/37, 78.4% *vs.* 3/37, 7.3%, *P* < *0.001*).

**Table 3:** Comparative analysis of CEUS characteristics of benign and malignant ductal lesions

Characteristics	Benign, n (%) (*n* = 44)	Malignant, n (%) (*n* = 38)	**χ** ^2^/t	*P*
No blood perfusion	3	1		
Blood perfusion	41	37		
Wash in			12.062	< 0.001^*^
Earlier	20(48.8)	33(89.2)		
Later	9(22.0)	0(0)		
Synchronous	12(29.3)	4(10.8)		
Wash out			1.417	0.492
Earlier	20(48.8)	22(59.5)		
Later	10(26.8)	9(24.3)		
Synchronous	11(24.4)	6(16.2)		
Enhancement intensity			15.386	< 0.001^*^
Hyper	18(43.9)	32(86.5)		
Hypo	12(29.3)	3(8.1)		
Iso	11(26.8)	2(5.4)		
Enhancement mode			5.701	0.017^*^
Homogeneous	22(53.7)	10(27)		
Heterogeneous	19(46.3)	27(73)		
Enhancement scope			40.592	< 0.001^*^
Enlarged	3(7.3)	29(78.4)		
Not enlarged	38(92.7)	8(21.6)		
Blood perfusion defects			12.947	< 0.001^*^
Presence	4(2.8)	17(45.9)		
Absence	37(90.2)	20(54.1)		
Peripheral high enhancement			24.28	< 0.001^*^
Presence	3(7.3)	22(59.5)		
Absence	38(92.7)	15(40.5)		
Radial-penetrating vessels			2.10	0.147
Presence	4(9.8)	8(28.6)		
Absence	37(90.2)	29(78.4)		
Boundary			18.56	< 0.001^*^
Clear	30(73.2)	9(24.3)		
Unclear	11(26.8)	28(75.7)		
Quantitative parameters				
AT	11.3 ± 7.01	8.27 ± 2.25	2.652	0.011*
k	2.2 ± 3.8	2.2 ± 0.87	0.021	0.984
TTP	26.1 ± 27.8	18.4 ± 11.7	1.261	0.111
PI	20.7 ± 82.4	10.7 ± 4.7	0.738	0.463
AUC	477.66 ± 364.46	760.38 ± 478.79	-2.95	0.040*
MTT	37.56 ± 25.74	30.78 ± 12.25	1.50	0.137

*AT* Arrival Time, *AUC* area under the curve, *k* Rising slope, *MTT* Mean Transit Time, *PI* Peak Intensity, *TTP* Time to Peak. * represents *P* < *0.05*

According to the results of quantitative analysis, the AT of the malignant group was shorter than that of the benign group (8.27 ± 2.25 *vs.* 11.3 ± 7.01, *P* = *0.011*), and the AUC was larger than that of the benign group (760.38 ± 478.79 *vs.* 477.66 ± 364.46, *P* = *0.040*) (Table 3).

### Logistic regression analysis

The variables (characteristics) with significant differences between the benign and malignant groups were selected as candidate variables, including shape, margin, inner echo, size, microcalcification, blood flow classification in conventional US and wash-in time, enhancement intensity, enhancement mode, enhancement scope, blood perfusion defects, boundary, peripheral high enhancement, AT, and AUC in CEUS.

Microcalcification (OR = 8.96, *P* = *0.047*) and an enlarged enhancement scope (OR = 27.42, *P* = *0.018*) were identified as independent risk factors for malignant ductal lesions on US and CEUS. (Table 4).

**Table 4:** Multivariate logistic regression of significantly different characteristics between benign and malignant ductal lesions

Characteristic	B	Standard error	Wald	*P*	OR	OR 95% CI	
						Lower	Upper
Shape	1.291	1.173	1.211	0.271	3.637	0.365	36.258
Margin	2.146	1.543	1.935	0.164	8.550	0.416	175.782
Inner echo	0.925	1.223	0.572	0.450	2.521	0.229	27.720
Size	0.837	1.354	0.645	0.352	2.102	0.273	24.725
Microcalcification	2.193	1.102	3.963	0.047*	8.962	1.034	77.653
Blood flow classification	0.999	1.044	0.916	0.339	2.714	0.351	20.985
wash in	-1.601	1.359	1.388	0.239	0.202	0.014	2.892
Enhancement intensity	1.280	1.053	1.478	0.224	3.598	0.457	28.346
Enhancement mode	-1.604	1.610	0.993	0.319	0.201	0.009	4.717
Blood perfusion defects	1.684	1.376	1.499	0.221	5.389	0.364	79.861
Enhancement scope	3.312	1.401	5.584	0.018*	27.428	1.759	427.620
Radial-penetrating vessels	-0.610	1.783	0.117	0.732	0.543	0.017	17.898
Boundary	0.747	1.194	0.391	0.532	2.111	0.203	21.930
Peripheral high enhancement	-0.003	1.468	0.000	0.998	0.997	0.056	17.707
AT	-.085	0.125	0.455	0.500	0.919	0.719	1.175
AUC	0.002	0.002	1.400	0.237	1.002	0.999	1.006
Constant	-13.183	5.513	5.717	0.017	0.000		

*CI* indicates confidence interval; and *OR* odds ratio

### Diagnostic performance analysis

**Fig. 5:**
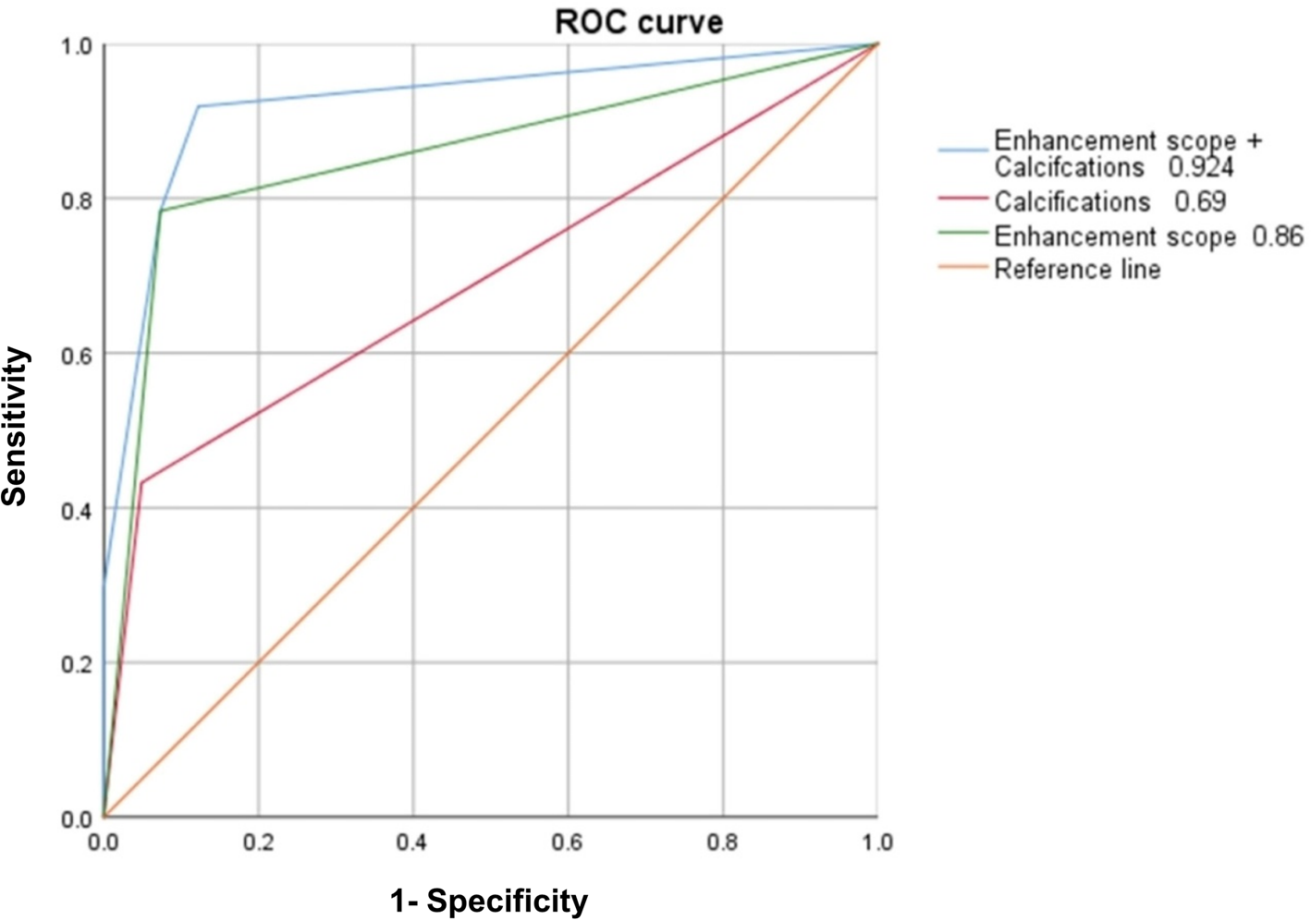
Receiver operating characteristic curve of enhancement scope, calcification and enhancement scope combined calcification. AUC_enhancement scope + calcifications_ *vs.* AUC_microcalcification_, *P* < *0.0005;* AUC_enhancement scope + microcalcifications_ *vs.* AUC_enhancement scope_, *P* = *0.007*; AUC_microcalcifications_ *vs.* AUC_enhancement scope_, *P* = *0.011*

Diagnostic performance was evaluated by using sensitivity (SEN), specificity (SPE), positive predictive value (PPV), negative predictive value (NPV), accuracy (ACC) and area under the receiver operating characteristic curve (AUC). The SEN, SPE, PPV, NPV, ACC, and AUC of microcalcification were 0.421, 0.955, 0.889, 0.656, 0.707, and 0.69, respectively; the enhancement scope (enlarged) was 0.784, 0.927, 0.901, 0.826, 0.859, and 0.86, respectively; and the microcalcifications combined with enhancement scope was 0.895, 0.886, 0.872, 0.907, 0.890, and 0.92, respectively (Table 5). AUC_enhancement scope + microcalcifications_ *vs.* AUC_microcalcification_, *P* < *0.0005*; AUC_enhancement scope + microcalcifications_ *vs.* AUC_enhancement scope_, *P* = *0.007*; AUC_microcalcifications_ *vs.* AUC_enhancement scope_, *P* = *0.011* (Fig. 5). In addition, CEUS was able to improve the BI-RADS classification of US (Supplementary materials, Tables 1 and 2). Furthermore, in benign lesions, 20 lesions (20/44, 45.5%) (intraductal papilloma) may avoid unnecessary resection, but follow-up observation if the proposed criteria (enhancement scope + microcalcifications) were applied.

**Table 5:** Receiver operating characteristic analysis of US and CEUS

Characteristic	SEN %	SPE %	PPV %	NPV %	ACC %	AUC	P	95% CI	
								Lower	Upper
Enhancement scope(enlarged)	78.4	92.7	90.1	82.6	85.9	0.86	< 0.001	0.764	0.947
Microcalcification	42.1	95.5	88.9	65.6	70.7	0.69	0.040	0.567	0.810
Enhancement scope combined Microcalcifications	89.5	88.6	87.2	90.7	89.0	0.924	< 0.001	0.859	0.988

*CI* indicates confidence interval, *SEN* sensitivity, *SPE* specificity, *PPV* positive predictive value, *NPV* negative predictive value, *ACC* accuracy, *AUC* area under receiver. AUC_enhancement scope + calcifications_ *vs.* AUC_microcalcification_, *P* < *0.0005*; AUC_enhancement scope + microcalcifications_ *vs.* AUC_enhancement scope_, *P* = *0.007*; AUC_microcalcifications_ *vs.* AUC_enhancement scope_, *P* = *0.011*

## Discussion

In the ACR BI-RADS ATLAS, “abnormalities of the duct” are called duct changes included in “Associated features” in BI-RADS, in which two concepts are described as follows: irregular or regular dilatation of duct(s) and dilated ducts with some echogenic intramammary ductal material [15]. Our study investigated how to identify malignant duct abnormalities using US and CEUS.

Advances in ultrasound, especially scanning with a high-frequency transducer, can be used to visualize clusters of microcalcifications that have a very high suspicion of malignancy [20]. *Soo *et al. [21] reported that, although suspicious microcalcifications are seen infrequently on sonography (23%), when detected, they are more frequently malignant than those seen on mammography alone. This is consistent with our study. In our study, microcalcification was more frequently detected in malignant lesions than in benign lesions (42.1% *vs.* 4.5%, *P* < *0.001*), which was strongly correlated with malignant duct lesions (OR = 8.96, *P* = *0.047*), showing that it was an independent factor predicting malignant ductal lesions on US. *Park *et al. [22] found that microcalcifications with associated ductal changes were the most common US finding of high-grade DCIS. Previous studies also reported that microcalcifications on ultrasonography were associated with poor pathologic results [23, 24]. The specificity of microcalcification was perfect (0.995), but the sensitivity was poor (0.421). One possible reason is that US is not sensitive enough for the detection of calcifications compared to mammography, but it can increase the specificity [20, 21]. Another reason is that not all malignant ductal lesions have microcalcifications, and US can help to identify noncalcified lesions, especially in patients with dense breasts. Moreover, *Moon *et al. found that malignant calcification is more frequently visualized on US than benign calcification, which may be obscured by echo of the breast parenchyma [20, 25]. Calcifications seen on US are more than three times more likely to be malignant than calcifications not seen on US [21, 25]. Although ultrasonography is not a standard technique for evaluating microcalcifications, it can give us more diagnostic confidence for malignant ductal lesions when they are observed on US.

In the development of breast cancer, angiogenesis is an important factor that regulates the growth and metastasis of tumors [16]. CEUS uses microbubbles as an intravascular tracer to evaluate and quantify tissue perfusion, and the enhancement only comes from blood vessels [16].

In our study, CEUS showed that 4 lesions (3 intraductal debris and 1 mucinous carcinoma) had no blood perfusion and were excluded from the qualitative and quantitative analysis of CEUS. The lesions without blood perfusion showed a simple intraductal mass with breast duct dilatation on US images. Intraductal debris is composed of intraluminal acidophilic material and foamy macrophages from real solid lesions. These benign lesions are seen more widely and cannot always be easily distinguished with US. When debris is mobile or in fluid form and does not show blood flow with any Doppler US, it can be easily identified [26]; however, when it is solidified and immobile, it can mimic intraductal papilloma [26]. Hence, it is easy to identify debris with CEUS, which shows no enhancement due to lack of blood supply and avoids unnecessary biopsies or advanced radiological imaging methods, such as MRI.

CEUS images showed blood perfusion in the remaining 78 lesions. Multivariate logistic regression analysis suggested that an enlarged enhancement scope (OR = 27.42, *P* = *0.018*) was an independent risk factor. The pathophysiologic basis is that malignant ductal lesions show infiltrative growth. Related studies have shown that angiogenic factors in malignant tumors promote angiogenesis and cause infiltration into surrounding tissues [27]. This leads to the formation of a chaotic microvascular network around the tumor, which will promote the growth of the tumor in all directions due to the anisotropy of malignant tumor growth. Hence, the enhancement area was enlarged compared with that on US. *Quan *et al. [28] reported that the enhancement scope expansion on CEUS may be the most useful indicator to identify malignant breast lesions. Several previous studies have also reported that an enlarged enhancement scope is an important finding to differentiate malignant lesions of the breast [17, 29–31]. *Drudi *et al. [16] concluded that the most specific sign of malignancy identified on CEUS was peripheral enhancement. The peripheral enhancement areas were generally outside the hypoechoic area of the lesion on conventional US (Fig. 4e), which is a special type of expansion of the enhancement scope. Several studies [16, 32, 33] reported that wash-out may indicate malignant lesions because of neoangiogenesis. In this study, we have evaluated the performance of wash out in the differentiation of benign and malignant lesions (Table 5). But there was no statistical significance between benign and malignant groups. The reason may be that breast ductal lesions, especially benign papillary lesions (intraductal papilloma) which are a kind of hypervascularity tumor pathologically composed of papillary projections with fibrovascular cores [34], which leads to wash-in/out rapidly of contrast medium. Previous studies [31, 35] have also shown that there was no significant difference about wash-out of contrast medium between benign and malignant papillary lesions which usually show ductal abnormalities on ultrasound.

For quantitative analysis, AT and AUC were not independent risk factors. This may be because malignant lesions have a large number of arteriovenous fistulas or chaotic microvascular networks, resulting in earlier AT of contrast medium and richer blood perfusion, making the AUC higher than that of benign lesions. However, intraductal papilloma is also a kind of hypervascularity tumor that is pathologically composed of papillary projections with fibrovascular cores [34]. Therefore, earlier AT and higher AUC were not independent risk factors for predicting malignant ductal lesions.

The AUC of microcalcifications combined with the enhancement scope was 0.92, which was higher than their AUC alone. Applying CEUS to BI-RADS classification can significantly improve the accuracy of classification, showing that CEUS is a good supplementary method in the diagnosis of ductal lesions.

Our study has some limitations. First, the sample size was relatively small. Second, the radiologists have some subjectivity when describing the morphologic characteristics. However, Kappa test showed that they had good agreement about the imaging analysis (kappa values = 0.81, *P* < *0.001*). Third, the inclusion criteria in the present study were based on the ultrasound characteristics. Other lesions presented as typical ductal abnormalities on ultrasound, which were not consistent with the final pathologic results and could have been mis-included. However, this diagnostic process indeed reflects real clinical work, in that physicians would have to appropriately manage those lesions. Therefore, this study could also provide guidance for radiologists. Finally, although ultrasonography is not a standard technique for evaluating microcalcifications, it can give us more diagnostic confidence for malignant ductal lesions when observed on US.

## Conclusion

The characteristics of benign and malignant ductal lesions are different. Microcalcification and enlarged enhancement scope were identified as independent factors for predicting malignant ductal lesions. The diagnostic performance can be significantly improved by the combined diagnosis, indicating that US and CEUS can be very useful in the differentiation of benign and malignant breast ductal lesions.

## Supplementary Information

**Additional file 1.**

## Data Availability

All data generated or analyzed during this study are included in this article.

## Abbreviations

ACR: American College of Radiology
AT: Arrival Time
AUC: Area under the curve
BI-RADS: Breast Imaging—Report and data system
CEUS: Contrast-Enhanced Ultrasonography
DCIS: Ductal carcinoma in situ
k: Rising slope
MG: Mammographic
MTT: Mean Transit Time
PI: Peak Intensity
ROC: Receiver Operating Characteristic Curve
TTP: Time to Peak
US: Ultrasonography
