# Supplementary material for: The diagnostic value of contrast-enhanced ultrasonography in breast ductal abnormalities
Source: Cancer Imaging. 2023 Mar 10;23:25. doi: 10.1186/s40644-023-00539-w (PMC10007791; doi:10.1186/s40644-023-00539-w)
Supplement: Supplementary file 1 — Additional file 1. [file 40644_2023_539_MOESM1_ESM.docx]

**Supplementary Material**

**1.** **Ultrasonography** **examination process**

The patients were asked to take the supine position to fully expose the breast. A comprehensive scan of the bilateral breasts was conducted. After the suspicious duct lesions were found, multisection and multiangle scanning was performed on the lesions. Meanwhile, color Doppler flow imaging (CDFI) was used to observe the blood flow. If calcifications were visible on US, a specimen radiograph was obtained to confirm their presence.

**2. CEUS** **examination process**

The mechanical index was set at 0.06, and the focus was placed at the bottom of the image. Before the contrast-enhanced ultrasound began, color Doppler US examinations were performed in different planes to evaluate peripheral and intralesional vascularity. The plane with the most blood flow was selected for CEUS. The selected plane included both the lesion and its surrounding normal tissue, if possible. After the US images of lesions were clear, the dual-screen CEUS mode was switched. Five milliliters of the contrast agent mixed with a saline solution was injected via an antecubital vein in a bolus fashion, followed by a flush with 5 mL of a normal saline solution. At the same time, dynamic images were recorded from the beginning of injection and observed for 180 seconds. The whole process was stored on the hard disk of the US machine in the form of video for further analysis.

**3. The Adler grades of the vascularity of the lesions on color Doppler US imaging**

Grade 0 represents no blood flow signals; grade 1 represents the spot or short line blood flow in the lesions; grade 2 refers to 3–4 punctate or short-line blood flow signals in the lesions, or one blood flow of which length is greater or equal to the radius of the lesions; and in grade 3, more than three stripe blood vessels are found in the lesions, which form a network.

**4. Definition of quantitative parameters of CEUS**

(a) Arrival time (AT; seconds) was defined as the time before the start of the rising time-intensity curve. (b) Time to peak (TTP; seconds) was defined as the time between the first arrival of contrast agent and the maximum signal intensity; (c) peak intensity (PI; dB) was defined as the maximum intensity of the time-intensity curve; (d) rising slope (k; dB/sec) referred to the speed of blood flow perfusion in the lesion area; (e) area under the curve (AUC) was defined as the area under the time-intensity curve that was proportionate to the total volume of blood in the region of interest; (f) mean transit time (MTT; seconds) was defined as the lesion’s enhancement duration.

**5. BI-RADS categories of ductal lesions on US and CEUS.**

The lesions were classified by two radiologists with more than 5 years of experience in US and CEUS diagnosis. When there were different opinions on classification, they discussed and reached an agreement. The classification of US was divided into the following five categories: 3, 4A, 4B, 4C, and 5 according to ACR 2013 ultrasound BI-RADS (5th edition). For CUES, the lesions were reclassified on the basis of US classification according to the results of this study. If the enhancement scope of the lesions was enlarged during CEUS, it would rise by 1 level based on the US classification; if there was no enhancement, it would be reduced by 1 level. In the rest of the cases, the classification remained unchanged.

**Table 1. BI-RADS categories of ductal lesions on US and CEUS.**

| BI-RADS | US | | CEUS | |
| --- | --- | --- | --- | --- |
|  | Benign (44) | Malignant (38) | Benign (44) | Malignant (38) |
| 3 | 10 | 2 | 13 | 2 |
| 4A | 20 | 10 | 26 | 2 |
| 4B | 11 | 12 | 4 | 10 |
| 4C | 2 | 8 | 1 | 17 |
| 5 | 1 | 6 | 0 | 7 |

**6.** **Diagnostic performances of US and CEUS**

Cases scored as BI-RADS 4B, BI-RADS 4C or BI-RADS 5 with a final histological diagnosis of a malignant lesion were considered true positives, while cases assessed as BI-RADS 3 or BI-RADS 4A with a final histological diagnosis of benign lesions were considered true negatives. Sensitivity, specificity, positive predictive value, negative predictive value and accuracy were evaluated for US and CEUS. A comparison of sensitivity, specificity, positive predictive value, negative predictive value and accuracy between US and CEUS was performed, and statistically significant differences were assessed with a two-tailed Fisher’s exact test. A *P* value of less than 0.05 was considered to be statistically significant (Table 2).

**Table 2. Comparison of the diagnostic performances of US and CEUS**

|  | US | CEUS | *P* |
| --- | --- | --- | --- |
| Sensitivity | 68.4% | 89.5% | *0.047* |
| Specificity | 68.1% | 88.6% | *0.036* |
| Positive predictive value | 65% | 87.2% | *0.034* |
| Negative predictive value | 71.4% | 90.7% | *0.028* |
| Accuracy | 68.3% | 89.0% | *0.002* |
